# Novel PEPA-functionalized graphene oxide for fire safety enhancement of polypropylene

**DOI:** 10.1088/1468-6996/16/2/025006

**Published:** 2015-03-27

**Authors:** Jia You Xu, Jie Liu, Kai Dan Li, Lei Miao, Sakae Tanemura

**Affiliations:** 1 School of Chemistry & Chemical Engineering, Guangzhou University, Guangzhou 510006, People’s Republic of China; 2 Key Laboratory for Renewable Energy, Guangzhou Institute of Energy Conversion, Chinese Academy of Sciences, Guangzhou 510640, People’s Republic of China; 3 Guangxi Key Laboratory of Information Material, Guangxi Collaborative Innovation Center of Structure and Property for New Energy and Materials, School of Material Science and Engineering, Guilin University of Electronic Technology, Guilin, 541004, People’s Republic of China

**Keywords:** graphene oxide, fire safety, polypropylene, PEPA, intumescent flame retardant

## Abstract

Polypropylene (PP) is a general-purpose plastic, but some applications are constrained by its high flammability. Thus, flame retardant PP is urgently demanded. In this article, intumescent flame retardant PP (IFRPP) composites with enhanced fire safety were prepared using 1-oxo-4-hydroxymethyl-2,6,7-trioxa-1-phosphabicyclo [2.2.2] octane (PEPA) functionalized graphene oxide (PGO) as synergist. The PGO was prepared through a mild chemical reaction by the covalent attachment of a caged-structure organic compound, PEPA, onto GO nanosheets using toluene diisocynate (TDI) as the intermediary agent. The novel PEPA-functionalized graphene oxide not only improves the heat resistance of GO but also converts GO and PEPA from hydrophobic to hydrophilic materials, which leads to even distribution in PP. In our case, 7 wt% addition of PGO as one of the fillers for IFRPP composites significantly reduces its inflammability and fire hazards when compared with PEPA, by the improvement of first release rate peak (PHRR), total heat release, first smoke release rate peak (PSRR) and total smoke release, suggesting its great potential as the IFR synergist in industry. The reason is mainly attributed to the barrier effect of the unburned graphene sheets, which protects by the decomposition products of PEPA and TDI, promotes the formation of graphitized carbon and inhibits the heat and gas release.

## 1. Introduction

Graphene is a two-dimensional material comprising a monolayer of sp^2^ hybridized carbon atoms arranged into a honeycomb lattice [1]. It is anticipated to be a promising material due to a variety of potential applications [2]. Recently graphene has shown great potential for fire safety applications of polymers [3]. The layered graphene material acts as a physical barrier which slows down the diffusion of gases and degradation products, and the interfacial interactions among graphene and polymers are the main reasons for the applications [4]. However, the use of graphene is limited by the lack of an effective method for large-scale production [5], by the strong tendency of graphene layers to stack [6] via van der Waals interactions and by their flammability. At present, the most promising strategies for the efficient preparation of large amounts of graphene sheets initially rely on the chemical conversion of graphite to graphite oxide [7, 8]. Graphite oxide has a layered structure with hydroxyl and epoxy groups on the basal planes and carboxylic and carbonyl groups at the graphene sheet edges [9]. Graphite oxide can be exfoliated in solution to form (monolayer) GO or partially exfoliated to form few-layer GO. However, the oxygen-containing groups and defects on GO make it thermally unstable [10], and the removal of these groups during reduction increases the tendency to agglomerate unless stabilized by decorated groups or polymers and surfactants [11, 12]. Generally, GO is exfoliated more readily than graphite, using thermal treatments or via sonication in water [13], because of the oxygen-containing groups. Afterwards, it can be decorated by different chemical groups capable of improving its thermal stability [14, 15] and enhancing its applicability. Furthermore, added chemical features are enhancing the possibility of interactions between the graphene and other chemical components. The presence of functional groups on GO surfaces has the additional benefits of easy dispersion in polar solvents or water, and it is the key factor in the physical and chemical properties of modified graphene [16]. More than that, these decorated graphenes provide opportunities for researchers to employ them in designing polymer nanocomposites [17, 18]. In the past few years, researchers have made successful attempts for GO and graphene–polymer composites [19–21]. Recently, a lot of attention from researchers has been attracted by modified GO for reducing fire hazards in polymer composites. Ye *et al* [22] prepared graphene–poly(methyl methacrylate) composites with improved flame retardant property. Tang *et al* [23] synthesized 2-amino-4,6-didodecylamino-1,3,5-triazine (ADDT) and covalently functionalized it onto graphene oxide nanosheets (GO–ADDT). It presents a better solubility in organic solvents, a significant increase in thermal stability and a greater residue formation in comparison to GO, which suggests the potential of GO–ADDT as a nanoadditive for polymeric systems. To the best of our knowledge, only a few studies have been reported on the generation of functionalized graphene derivatives between char-forming agent and graphene oxide, such as phenyl dichlorophosphate, which enhances the flame retardancy of epoxy resin [2]; 9,10-dihydro-9-oxa-10-phosphaphenanthrene-10-oxide (DOPO), which proved that the synergetic effect of DOPO–rGO is quite useful [24]. Therefore, it is very important to obtain highly flame retardant functionalized graphene derivatives, which disperse in nonpolar organic solvents.

**Figure 1.**
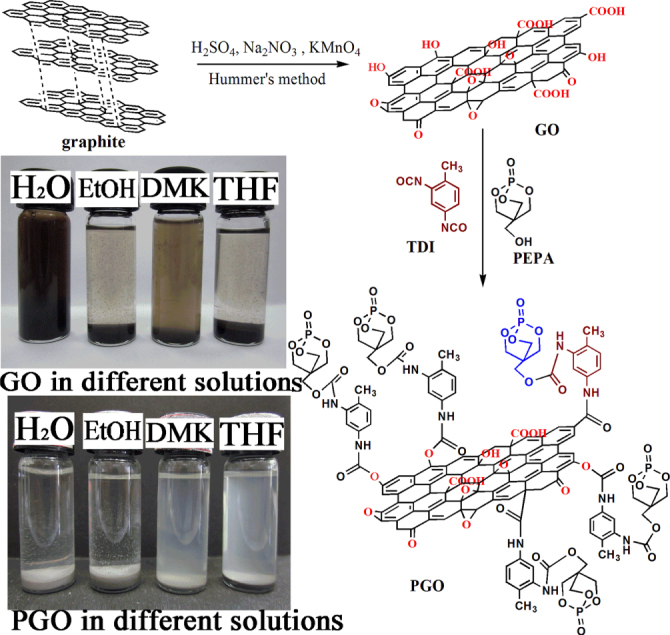
Schematic illustration of the synthesis route for PGO and digital photos of GO, and PGO (500 mg) dispersions in H_2_O, EtOH, DMK and THF (4 ml) after standing for 2 h.

Polypropylene (PP) is easily ignited and burned, and improving its fire safety properties is highly desirable due to the huge applications of PP [25]. Intumescent flame retardants (IFRs) comprise three basic elements which are called dehydrating agent [26], char-forming agent and foaming agent. And the IFRs are preferred due to their environmental friendly property [27]. A caged structure organic compound, namely 1-oxo-4-hydroxymethyl-2,6,7-trioxa-1-phosphabicyclo [2.2.2] octane (PEPA) [28], is a high-performance environment friendly char-forming agent for fire-retardant PP. It can be spontaneous or catalytic materials to form a molten char layer (equation (1)) at the surface of polymer materials [29, 30] under combustion. The molten char layer acts as a protective coat. However, there is a hydroxyl group in its structure, which makes it hydrophilic. The hydrophilic properties of PEPA make the flame retardant PP composites moisture sensitive, which will decrease flame retardancy because of the exudation of the additive [31]. To reduce the hydrophilicity and raise flame retardancy of GO and PEPA, we present a scheme for the covalent attachment of PEPA to GO nanosheets by the intermediary agent, toluene diisocynate (TDI) (figure 1), which is a perfect medium for the char-forming agent [32, 33]. It is expected that PEPA and TDI could form a molten char layer on the surface of PGO under the fire, which prevents graphene from burning out, and then the graphene may act as a physical barrier to protect the underlying polymers.

In this paper, GO was successfully decorated by PEPA via a mild chemical reaction, and added it into intumescent flame retardant PP (IFRPP) system to enhance thermal stability and drop off combustion performance of PP, by the 70 s delay of first release rate peak (PHRR), 5 MJ m^−2^ reduction of heat release rate peak (PHRR), 40 and 34% reduction of the first smoke release rate peak (PSRR) and total smoke release (TSR), respectively. Equation (1) shows the role of PEPA in the IFR system
1
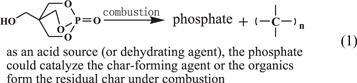



## 2. Experimental

### 2.1. Materials

Natural graphite powder was purchased from Sinopharm Chemical Reagent Co. Ltd (Shanghai, China). PEPA was synthesized according to [34]. Concentrated sulfuric acid (98%), sodium nitrate, potassium permanganate, 30% H_2_O_2_ solution, hydrochloric acid, TDI, dimethyl formamide (DMF), dichloroethane (EDC), absolute ethyl alcohol (EtOH), dimethyl ketone (DMK) and tetrahydrofuran (THF) were all of analytical reagent grade and provided by Guangzhou Chemical Reagent Factory (Guangzhou, China). IFR as described below, was mainly composed of ammonium polyphosphate.

### 2.2. Preparation of GO and PGO

GO was obtained by oxidation of natural graphite flake based on a modified Hummers method [34, 35]. The highly oxidized layers of GO could disperse in DMF with long-term stability (three weeks) [16]. GO was exfoliated into layered structure sp^2^ hybridized carbon and dispersed in the DMF solution by ultrasonic treatment to obtain a superior diffusion of reactive species.

A suspension of as-prepared GO (300 mg) in DMF (100 ml) by ultrasonication (60 min, room temperature) and TDI (15 ml) were added to a three-neck flask (250 ml) with stirring (12 h, room temperature). Afterwards, PEPA (10 g) was added into the mixture with stirring (5 h, 60 °C). The product was poured into a beaker (500 ml), then EDC (200 ml) was added into the mixture, a gray precipitate separated out. The precipitate was collected by filtration and dried in a vacuum oven at 80 °C overnight. Figure 1 illustrates the synthesis of GO and PGO.

### 2.3. Preparation of PP/IFR/PGO nanocomposites

Well dried PGO, IFR, and PP were mixed in a certain mass ratio (table 2); the mixture was extruded into pellets in a twin screw extruder (BL-6175-A, Dongguan, China) at 195 °C. Then well dried pellets were added into the injection-molding machine (HAITIAN SA600/100, Ningbo, China) and molded into standard testing bars for further testing.

### 2.4. Characterization

Fourier-transform infrared (FTIR) measurements were performed on a Nicolet IS-10 spectrometer, and samples were prepared as potassium bromide pellets. X-ray photoelectron spectroscopy (XPS) measurements were carried out on a MultiLab 2000 (Thermo Electron Corporation, England) with a 14.9 keV Al Ka x-ray source. Raman spectra were collected with a LabRam HR800 UV Raman microscope (Horiba Jobin-Yvon, France), using an Ar+ ion laser with excitation wavelength of 514 nm. Thermogravimetric analysis (TGA) was performed in nitrogen atmosphere with a Perkin-Elmer TGA 2050 instrument at a heating rate of 20 °C min^−1^. Morphology analyses of GO and PGO were carried out on a JEOL JEM-2100 transmission electron microscope (TEM). Scanning electron microscope (SEM) images were obtained on a JEOL JSM-6380LV SEM.

### 2.5. Fire test

Cone calorimeter test (CCT): samples were exposed to a Fire Testing Technology cone calorimeter with an external heat flux of 50 kW m^−2^ according to ISO 5660-1. The size of the samples was 100 × 100 × 3.2 mm^3^. The data presented in the following are averages from at least three experiments.

Limiting oxygen index (LOI) test: it is a standard test method for measuring the minimum oxygen concentration to support candle like combustion of plastics. LOI was measured using an HC-2 oxygen index instrument on 100 × 6.5 × 3.2 mm^3^ rods according to the standard ASTM 2863/77.

UL-94 test: vertical burning tests were carried out using a CZF-2 vertical burning test instrument with sheet dimensions of 127 × 12.7 × 3.2 mm^3^ according to ASTM D-635-77.

## 3. Results and discussion

### 3.1. Characterization of GO and PGO

The synthesis route of PGO and digital photos of GO and PGO (500 mg) dispersions in H_2_O, EtOH, DMK and THF (4 ml) are illustrated in figure 1. It is well known that GO can be easily exfoliated and well dispersed in water due to its hydroxyl, carboxyl and epoxide groups. The caged structure organic compound, PEPA, is also a hydrophilic substance (figure S1). After a suitable ultrasonic treatment, GO can produce stable dispersions in water exhibiting a light yellow color (figure 1). However, it’s obvious that PGO could not be well dispersed in H_2_O and EtOH, and it changed into gray instead of light yellow. Meanwhile, the PGO can form stable colloidal suspensions in DMK and THF due to the reduction of the hydroxyl and epoxide groups, which enhances the interface interaction between the organic groups and the organic solvent.

**Figure 2.**
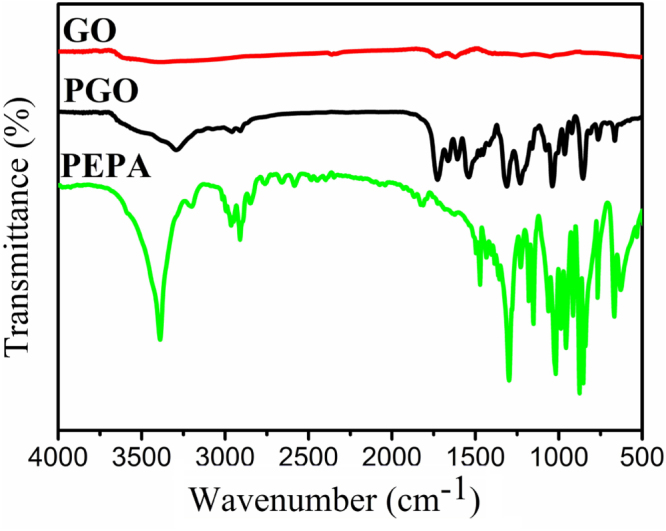
FTIR spectra of GO, PGO and PEPA. The curves of GO, PEPA, and PGO are offset vertically by 0.55, −0.5, and −0.23%, respectively.

The chemical components of GO and PGO were studied by FTIR and XPS. In the FTIR spectra (figure 2), the GO curve shows the characteristic absorption of GO: −OH at 3389 cm^−1^, C=O at 1718 cm^−1^, C=C and the hydroxyl in the adsorbed water at 1617 cm^−1^ and C–O–C at 1000–1300 cm^−1^ [4]. The structure of PEPA is confirmed with characteristic absorption at 3393 cm^−1^ (−OH stretching absorption), 2968 cm^−1^, 2908 cm^−1^ (−CH_2_−), 1295 cm^−1^ (P=O), 1017 cm^−1^, 952 cm^−1^ (P–O–C), and 854 cm^−1^, 761 cm^−1^, 663 cm^−1^, 625 cm^−1^ (typical of caged structure) in the FTIR spectra (figure 2), which is consistent with previous report [36]. The main peaks of GO and PEPA could be observed at the spectra of PGO, and the peaks at 3078, 1608, 1540 and 1475 cm^−1^ is attributed to benzene ring. It demonstrates the actual occurrence of the chemical reactions among GO, PEPA, and TDI, in addition the caged structure of PEPA does not been destroyed in the reactions.

**Figure 3.**
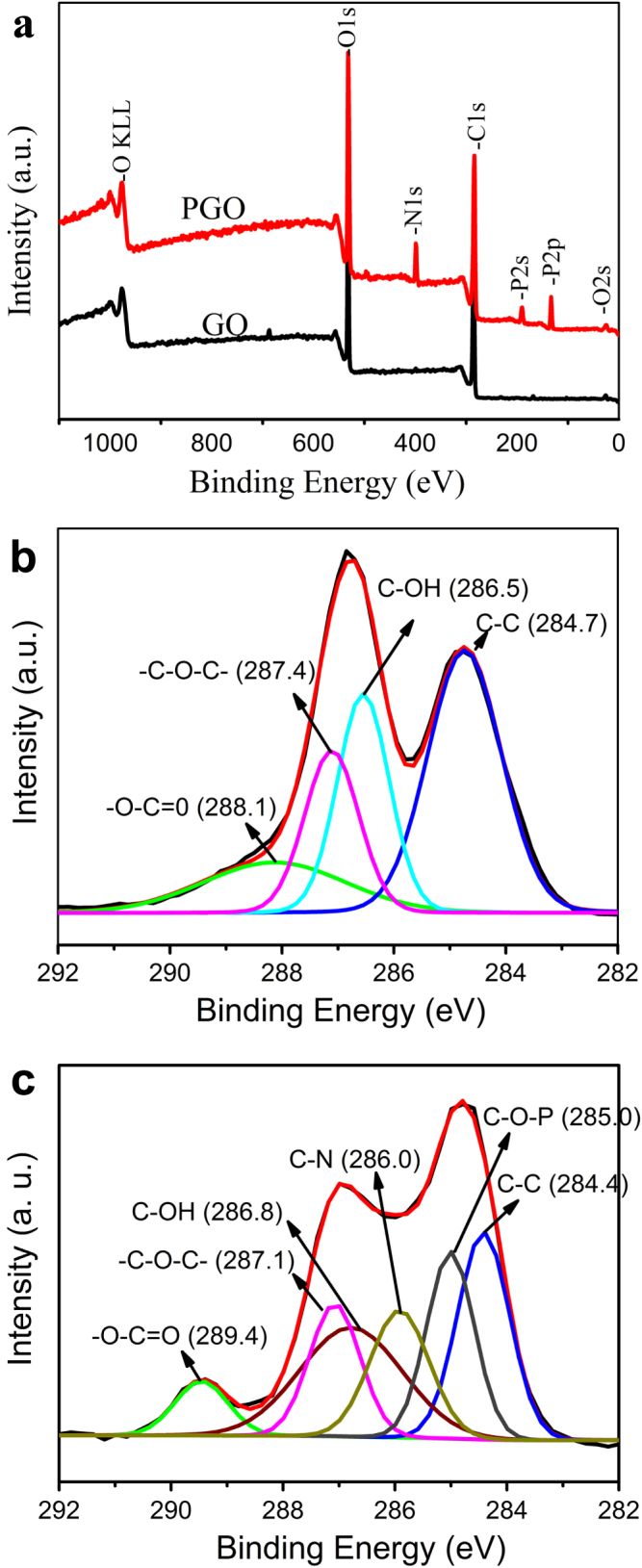
XPS spectra of GO and PGO (a), higher resolution C1s spectra of GO (b) and PGO (c). The curves of PGO (a) is offset vertically by 10 000 a.u.

After modification, the removal of oxygen groups and the formation of chemical bonds on the surface of GO were confirmed by XPS spectra. Figure 3 shows the XPS spectra of GO and PGO in a binding energy range of 0–1100 eV (figure 3(a)), and C1s scan of GO and PGO (figures 3(b) and (c)). As shown in figure 3(a), some peaks are observed at around 189.9 eV (P2s), 285.2 eV (C1s), 399.6 eV (N1s) and 531.1 eV (O1s). But only two peaks are observed at around 285.2 eV (C1s) and 531.1 eV (O1s) in the spectrum of GO, which indicates that PGO contained oxygen, nitrogen, phosphorus, and carbon. The C1s scan of GO and PGO (figures 3(b) and (c)) display the presence of four kinds of carbon in the GO and PGO: C=O, C–O–C, C–OH and C–C in the graphitic domain, and the peaks of C–O–P and C–N appear in the curve of the PGO. In comparison, the C–O–C signal is increased after the functionalization, however, the C–OH is decreased, which indicates the C–OH on the surface of the GO is reduced into C–O–C in the process of functionalization reaction.

**Figure 4.**
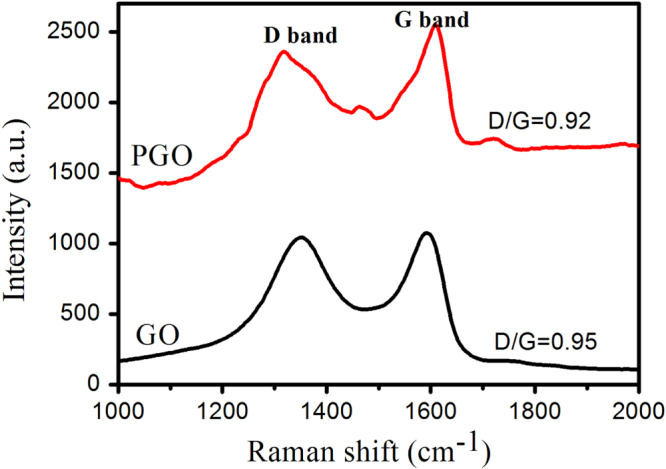
Raman spectra of GO and PGO.

Raman spectra (figure 4) show the peak positions and the ratio between the peak intensity of D band and that of G band (D/G) for GO and PGO, which reflects the structural changes occurring during the chemical processing from GO to PGO. D band (at about 1355 cm^−1^) is a breathing model of *κ*-point phonons of A_1g_ symmetry, G band (at about 1600 cm^−1^) is usually assigned to the E_2g_ phonon of C sp^2^ atoms [37]. As in figure 4, the Raman spectrum of GO is similar to that of PGO, suggesting that the skeletal structure of GO remains in the PGO after functionalization. However, there is a slight decrease of the D/G intensity ratio after functionalization, the decrease of the ratio indicates an increase of topological disorder in the graphene layer and a decrease in size of nanocrystalline graphene.

**Figure 5.**
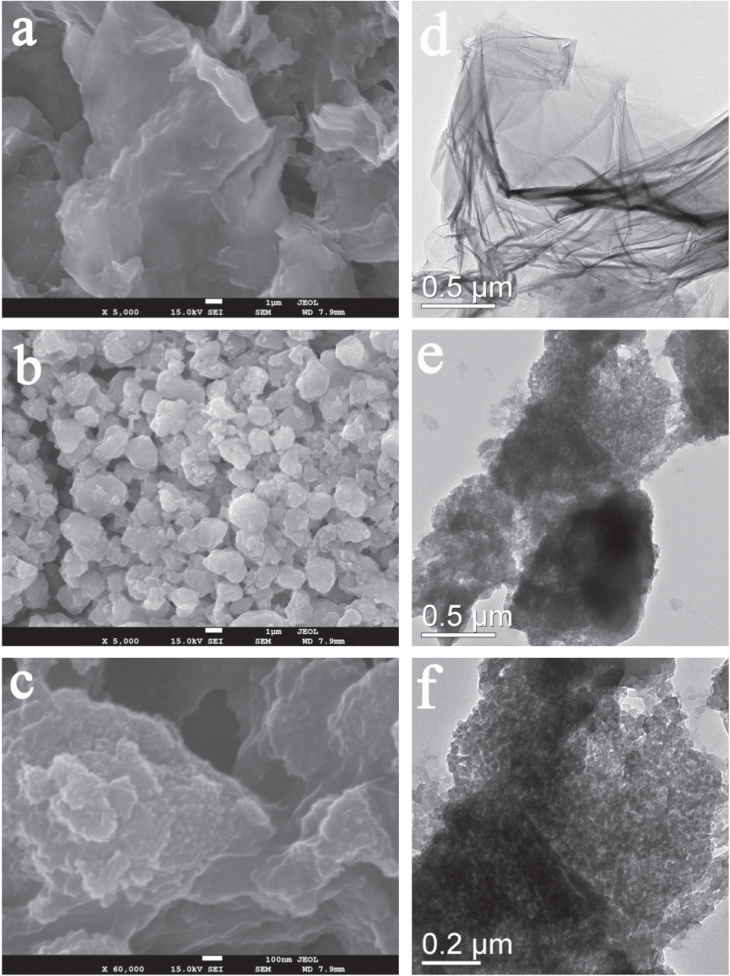
The SEM images of GO (a), PGO (b), (c) and TEM images of GO (d), PGO (e), (f).

SEM and TEM images are used to reveal the surface and structure morphology of GO and PGO at the submicrometer scale in figure 5. The SEM and TEM images of GO display that GO sheets exhibit a typically flat yet wrinkled nano-platelet shape within several ten-of-microns size (figures 5(a) and (d)) after an appropriate ultrasonic treatment. After surface functionalization, the ordered flat structure was broken into many disordered small pieces within several microns size (figures 5(b) and (c)), and there are many small dots wrap around the cracked GO sheets, as shown in figures 5(c), (e) and (f). These evidences demonstrate that the chemical reaction of the organics and –OH on the GO sheets could fracture the sheets due to the large size organic bounds planted into the narrow space of the layers. The strong inter- and intra-molecular interactions [38] make GO crushed and reassembled. During these processes the PEPA dots grow on the GO sheets and assemble into the structure as shown in figure 5(c). The PEPA, a kind of flame retardant additive, that cladding on the GO sheets is expected to form a protect layer on fire, which avoids the GO from burning out.

**Figure 6.**
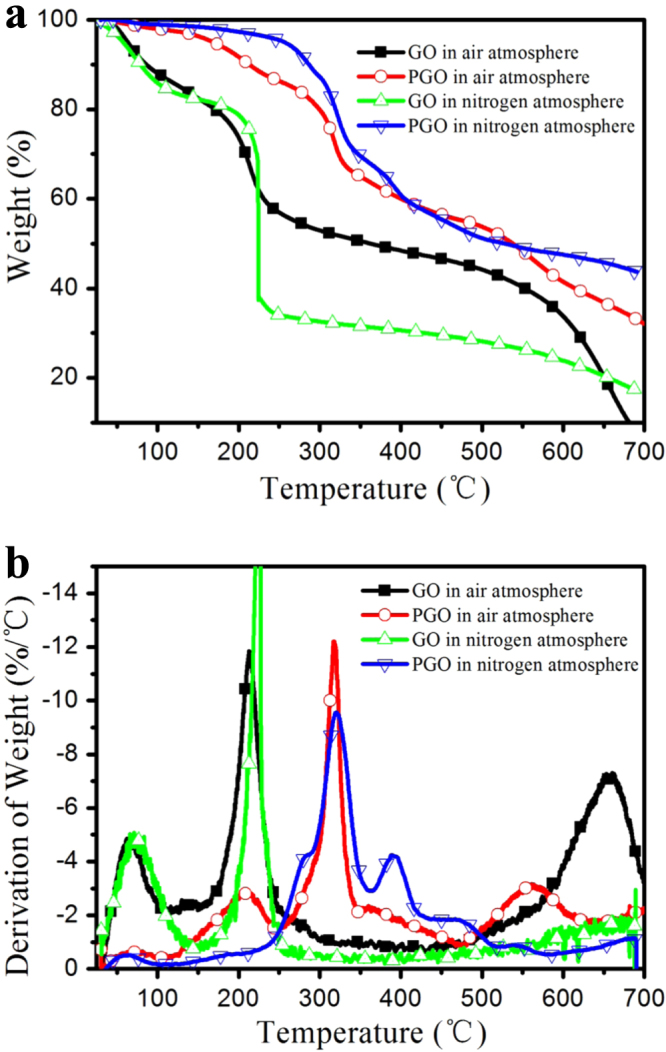
TGA (a) and DTG (b) patterns of GO and PGO in air and nitrogen atmosphere.

The functionalization and reduction of GO improved the thermal stability of GO [39–41]. Figure 6 shows TGA and DTG patterns of GO and PGO in nitrogen and air atmosphere, respectively. As figure 6 shows GO is thermally unstable with the first peak from 50 to 100 °C due to the removal of water, and its main mass loss of 65% at around 225 °C, which belongs to the decomposition of the functional groups (–OH and –COOH) and the graphene structure. The PGO has the first peak as well as GO from 50 to 100 °C in nitrogen atmosphere, but the peak intensity is much lower than that of GO, it demonstrates that the functionalization process reduces the hydroscopicity of GO, which is consistent with the results of the solubility studies mentioned above. In contrast, the thermal decomposition of PGO is divided into two steps (320 and 390 °C) in figure 6, which are much higher than that of GO, and exhibit much less mass losses than that of GO. The TGA results of GO and PGO in nitrogen or air atmosphere are consistent, except for behavior above 500 °C, which might be the oxidation of carbon materials. The significant enhancement of the thermal stability of GO is due to the decoration of PEPA and TDI on the surface of GO, they decompose first around 320 °C, and the cross-linked residual carbon of the decomposition covers on the surface of GO sheets could protect it from decomposing at this temperature. The improvement of the thermal stability of PGO is beneficial for its application as a flame retardant nano-filler.

### 3.2. Dispersion of PEPA in flame retardant PP composites

**Figure 7.**
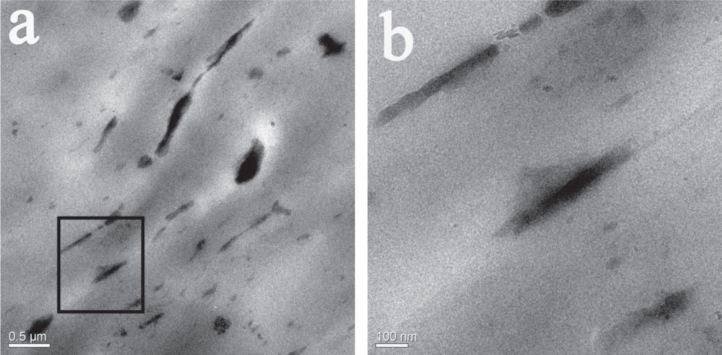
TEM image of 7 wt% PGO loaded in IFRPP composite (a), enlarged TEM image of (b).

As a polymer additive, PGO is supposed to act as a char-forming agent as well as barrier preventing the transfer of combustion gases to the flame zone and energy feedback, thus the evenly distributed of the fillers in matrix is necessary. TEM images shown in figure 7 are IFRPP composite containing 7 wt% PGO powder, which characterize the distribution of the PGO in PP matrix. It is found that the layered graphene sheets with a high aspect ratio showing the same orientation arrangement and no large aggregation, which demonstrates the good dispersion of PGO in IFRPP composite. It could be attributed to the elimination of polar functional groups, which enhances the interfacial interactions among graphene and PP. And the layered graphene material, shown in figure 7, could act as a physical barrier which slows down the diffusion of gases and degradation products.

### 3.3. Thermal and flammability properties of flame retardant PP composites

**Figure 8.**
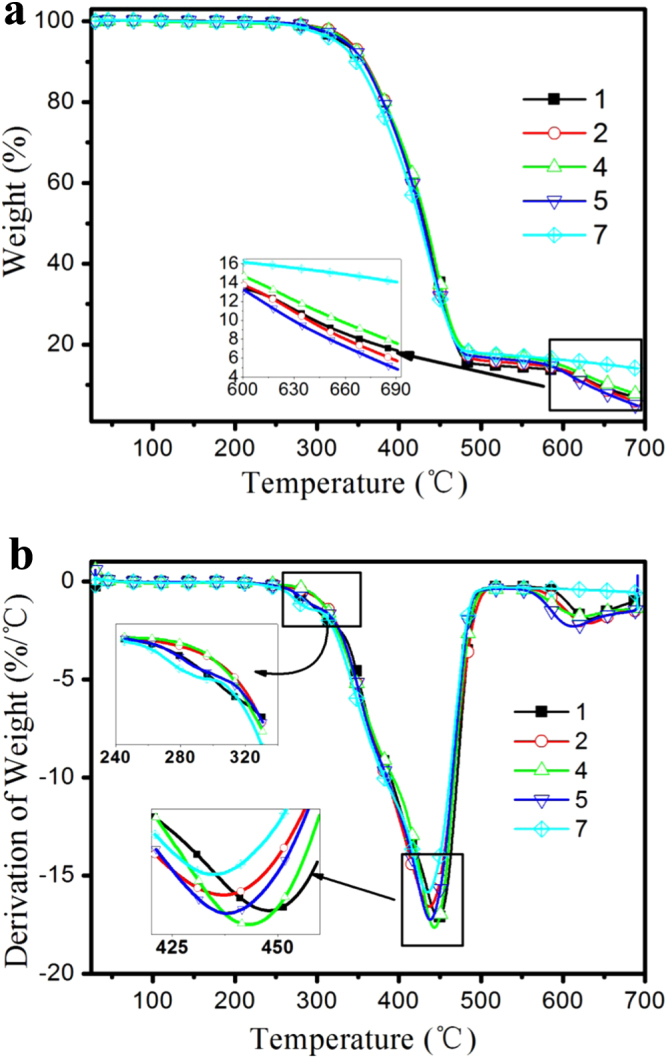
TGA (a) and DTG (b) of IFRPP composites.

TGA and DTG curves of flame retardant composites are shown in figure 8 and their data are listed in table 1. The thermal degradation processes of the samples are similar, but there are still some differences. There is a small decomposition peak at about 280 °C based on the DTG profile (figure 8(b)) of sample 7, which might be due to the decomposition of carboxyl and hydroxyl that creates water. The *T*_max_ of the IFRPP with 7 wt% PEPA or PGO are 438 and 434 °C, respectively, which are lower than that without one, since PEPA and PGO is thermally unstable compared to IFRPP composite and its major mass loss occurs below 350 °C due to the decomposition of the oxygen-contained functional moiety. It is worth to mention that the *R*_max_ of sample 7 decreases as well, owing to the multifunctional groups of PEPA and TDI, which could form a crosslinking char layer during heating or burning and covers on the surface of graphene sheets, protecting the graphene from burning out. However, the *R*_max_ value of sample 5 is the highest, even higher than that of sample 1, which is due to the high thermal conductivity of GO [42] as well as the high barrier layer material [43, 44]. If GO is insufficient in IFRPP composite, its thermal conductivity effect would prevail instead of the barrier effect, which results in the rapid decomposition of the IFRPP material. When GO is sufficient in IFRPP composite it would present an excellent flame retardant effect, as the curve for sample 7 in figure 8(a) and table 1, which exhibits a lowest *R*_max_ value and a maximum char yield. The results indicate that the PGO is much better than that of PEPA in terms of delaying the thermal degradation of flame retardant PP and enhancing the char formation.

**Table 1.** TG data of flame retardant composites in N_2_.

Sample	Composition	*T*_onset_ (°C)	*T*_max_ (°C)	*R*_max_ (%)	Residue (%)
1	70%^a^ PP/30%IFR	330	448	17.16	6.80
2	70% PP/27%IFR/3%PEPA	339	442	17.65	5.71
4	70% PP/23%IFR/7%PEPA	336	438	17.25	7.55
5	70% PP/27%IFR/3%PGO	333	437	16.60	4.81
7	70% PP/23%IFR/7%PGO	324	434	15.86	14.07

70%^a^ in this table they refer to weight percentage. *T*_onset_ corresponds to the temperature at the 5 wt% weight loss. *T*_max_ corresponds to the temperature of the maximum weight loss rate. *R*_max_ corresponds to the decomposition rate at *T*_max._

LOI measurements and UL-94 vertical burning tests are widely used to evaluate the flame retardant properties of materials [45]. According to the results of table 2, the material without PEPA and PGO is no level in the UL-94 test and only with an LOI value of 21. When the addition of PEPA or PGO in IFRPP composites is 3 wt%, its LOI value increased to 23.5 and 24, respectively, UL-94 test increased to V-2 and V-0, respectively. LOI value of IFRPP composites increases as the additional of PEPA or PGO increases, the UL-94 test has the same trend. But the same addition of PEPA and PGO have the uniform trend of LOI and UL-94 test, which demonstrates that the presence of GO has no contribution to the results of the flame retardant tests.

**Table 2.** The LOI and UL-94 test of the flame retardant PP.

			UL-94 test		
Sample ID	Composition	LOI test (vol %)	Rate	Dripping	Ignite the absorbent cotton
1	70% PP/30%IFR	21	NR	Yes	Yes
2	70% PP/27%IFR/3%PEPA	23.5	V-2	Yes/No^a^	No
3	70% PP/25%IFR/5%PEPA	26	V-0	No	No
4	70% PP/23%IFR/7%PEPA	27	V-0	No	No
5	70% PP/27%IFR/3%PGO	24	V-0	No	No
6	70% PP/25%IFR/5%PGO	26.5	V-0	No	No
7	70% PP/23%IFR/7%PGO	27	V-0	No	No

a Yes/No correspond to the first/second flame application.

**Figure 9.**
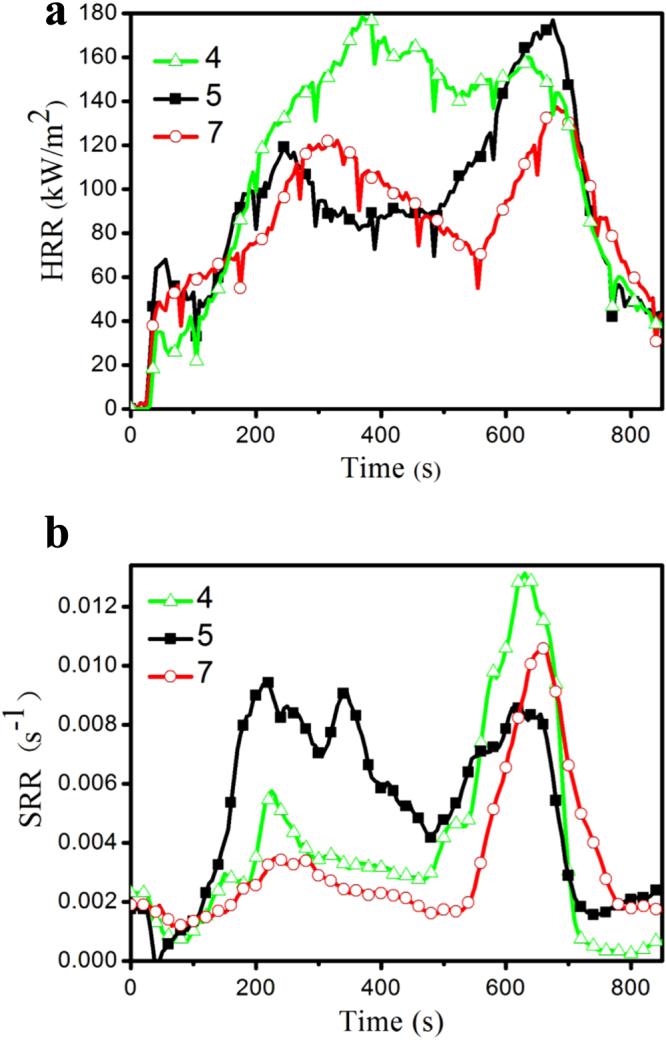
The HRR (a) and SRR (b) curves of CCT.

CCT analysis is a powerful tool to evaluate the flame retardant characteristics of flame retardant materials. It has good correlation with real fire disaster and is used to predict the combustion behavior of materials in real fires [46–48]. CCT is generally used to determine the HRR, total heat release (THR), SRR and TSR. Figure 9 demonstrates the HRR and SRR curves of samples 4, 5, and 7 at a heat flux of 50 kW m^2^, respectively. Figure S2 represents the THR and TSR of the IFRPP composites.

HRR and SRR have been found to be the most important parameters to evaluate fire safety [49]. As shown in figure 9(a), the HRR curves of IFRPP composites show two peaks. Sample 4 burns very fast after ignition, shows a maximum HRR peak with a value of 178 kW m^−2^ at 371 s and lasts for 270 s. In the case of sample 5 and sample 7, the maximum peaks come at about 675 s with the values of 174 and 136 kW m^−2^, respectively, and the first HRR peak of sample 7 comes later than that of sample 5. It indicates that PGO is a perfect char-forming agent for enhancing the fire safety of PP by prolonging the arrival time of the combustion peak and reduced the HRR and THR (figure S2(a)), which is due to the unburned GO sheets that are thought to possibly form a continuous, protective char layer that acts as a thermal insulator and a mass transport barrier [50]. The char layer is strong enough to resist heat and oxygen from the cone calorimeter as described in section 3.4 and prevent material from cracking as well as pursuing the further degradation. Figure 9(b) shows the SRR curves of samples 4, 5 and 7. It shows that the time of maximum smoke release for sample 7 is also decreased. However, the onset release smoke volume of sample 5 is much larger than that of the other two samples. TSR curves of samples 4, 5, 7 (figure S2(b)) have the same trend. That is because sufficient GO sheets could form an intact char residue acting as a physical isolation, however, the lack of GO sheets displays high thermal conductivity instead of physical isolation.

### 3.4. Analysis of char residue after cone calorimetric measurement

**Figure 10.**
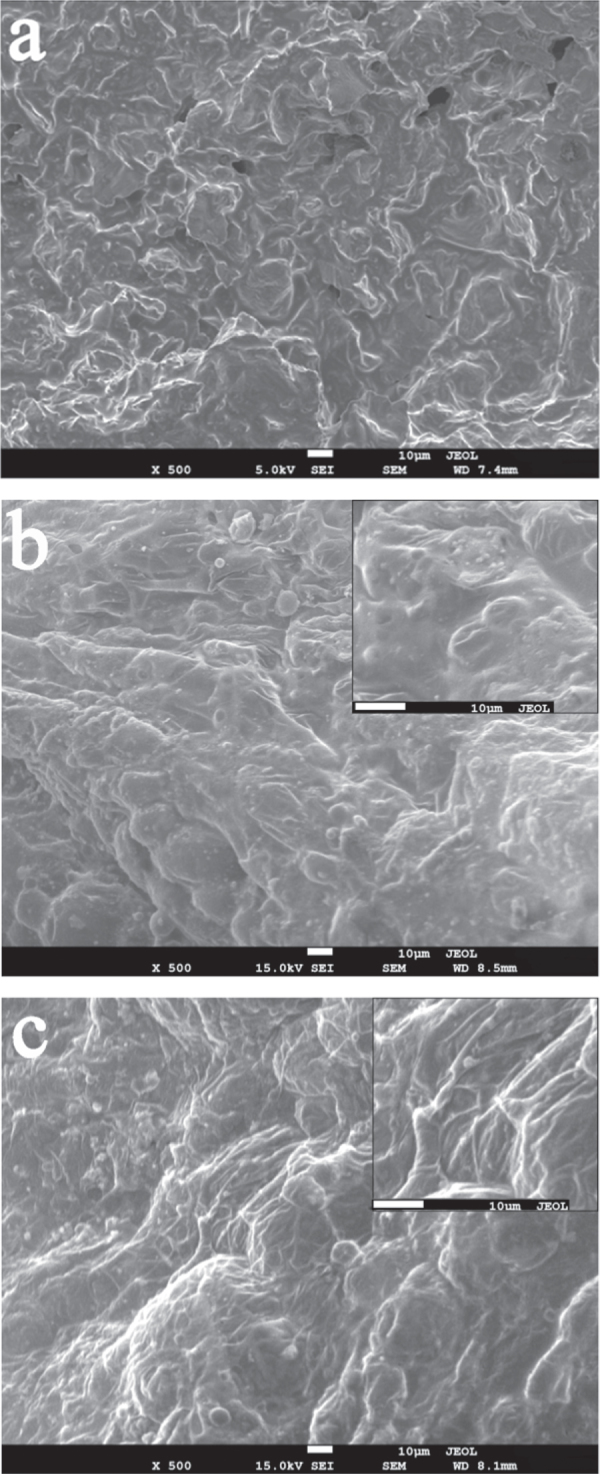
The SEM images of intumescent char layer: sample 1 (a), sample 4 (b), sample 7 (c).

The morphology of the residue chars of IFRPP composites after CCT is shown in SEM images in figure 10. The samples with PEPA and PGO produced a continuous, intact and intumescent outer surface, which acts as an insulating barrier, prohibits the oxygen and heat from reaching the underlying substrate due to polyphosphoric acid (decomposition products of PEPA or PGO). Sample 7 is more compact with fewer bubbles appearing on its surface than that of sample 4, suggesting that it could form a better barrier cross-linked network structure through lamellar structure of GO sheets to develop a more compact char layer with better physical barrier ability and mechanical performance.

**Figure 11.**
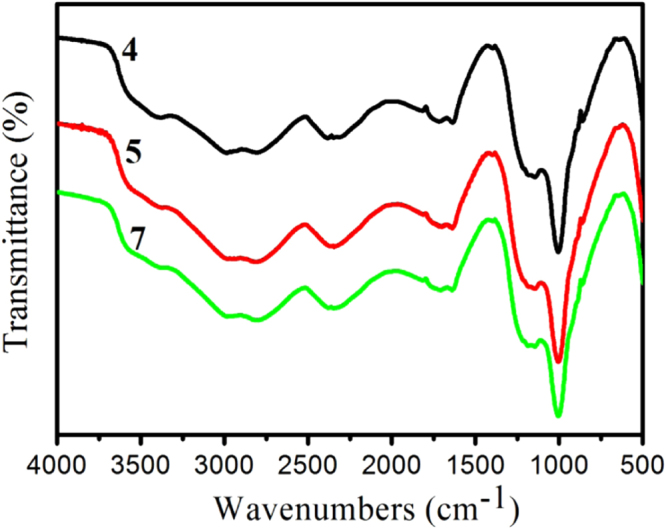
FTIR spectra of the residual char of IFRPP composites. The curves of samples 4, 5, and 7 are offset vertically by 0.45, 0.2, and 0%, respectively.

**Figure 12.**
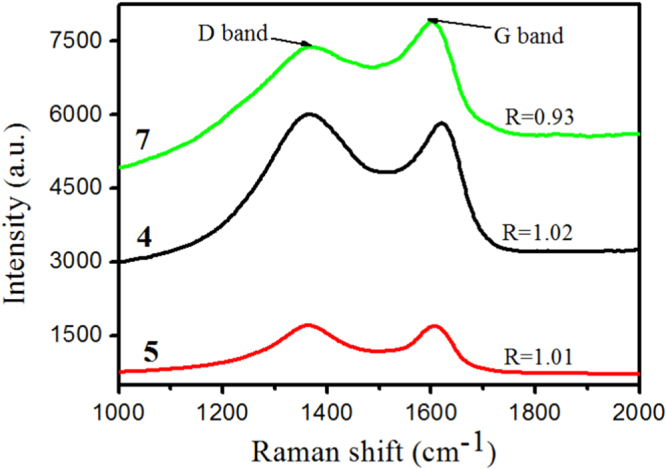
Raman spectra of residual char for IFRPP composites.

To clarify the cross-linked network char residue formation mechanism at the intumescent outer surface, FTIR (figure 11) and Raman (figure 12) spectroscopies were employed to characterize the residue char for samples 4, 5 and 7. As shown in FTIR results, there is no clear distinction among the samples with and without GO. The peaks around 1630 cm^−1^ correspond to the stretching vibration of phenyl, indicating the formation of polyaromatic species or graphitic structures during combustion [25]. The absorption bond at 1149 cm^−1^ is attributed to P–C–O structure in P–C complex, and the peak around 1000 cm^−1^ is for the symmetric vibration of P–O bond in P–O–C group [51]. Based on FTIR analysis of residue chars, the chemical structures of all samples are basically similar.

Raman spectroscopy offers a powerful tool for characterizing carbonaceous materials [10]. Figure 12 shows the Raman spectra of the residual char of IFRPP composites obtained from CCT. The samples exhibit a similar shape, with two peaks at around 1610 and 1369 cm^−1^. As mentioned in section 3.1, the two characteristic bands (D bands at 1369 cm^−1^ and G bands at 1610 cm^−1^) could evaluate the order degree of carbon materials: D band associated with the unorganized carbon structure and G band related to the organized carbon in the graphitic structure [30]. Furthermore, Tuinstra found that the relative peak intensity ratio (*R*) of the D peak to the G peak is inversely proportional to an in-plane microcrystalline size and/or an in-plane phonon correlation length obtained from Raman spectroscopy [52]. Therefore, *R* is a good parameter to state the graphitization degree of carbon materials, the higher the value of *R*, the lower the graphitization degree of chars. The *R* values of samples 4, 5 and 7 are measured (figure 12) and found to be 1.02, 1.01 and 0.93, respectively. The values for samples 4 and 5 do not differ much, which are in agreement with the results of CCT, and demonstrate that the lack of GO sheets could not play the role of physical isolation, in the case of fire it could not form the continuous structured graphitic structure. The minimum *R* value of sample 7 illustrates that the production of structured graphitic structure of sample 7 is increased, therefore, the perfect thermal insulator, mass transport barrier and mechanical performance of char layer has been formed.

## 4. Conclusions

In this article, a caged structure organic compound, PEPA, was successfully introduced onto the surface of GO sheets by using TDI as the medium. The changes of the structure, chemical composition, morphology and functionalities of the new materials were investigated by various techniques. The results confirmed that PEPA and TDI were successfully grafted onto the surface of the GO sheets, and PGO properties changed from hydrophobic to hydrophilic. TGA revealed that the functionalization of PEPA and TDI could enhance the thermal stability of GO due to the reduction of the oxygen-containing groups and the build-up of strong interactions between the organic molecular fragments and GO. The thermal and combustion properties of the PGO–IFRPP composites have also been studied. The results stated that the addition of PGO could not benefit to the value of the flame retardant tests compared to the PEPA, but it could reduce the fire hazards of the polymer materials by markedly reducing the HRR, THR, SRR and TSR values as the CCT results presented. The reasons should be that PEPA and TDI (on the surface of GO) decomposed first and formed a crosslinking char layer (due to the formation of polyphosphoric acid (decomposition products of PEPA)) covering on the surface of the GO sheets to protect GO sheets from burning. Consequently the abundant unburned GO sheets could take part in forming a compact char layer with superior physical barrier ability, mechanical performance and thermal stability.
